# Simultaneous Pickup and Delivery Traveling Salesman Problem considering the Express Lockers Using Attention Route Planning Network

**DOI:** 10.1155/2021/5590758

**Published:** 2021-05-22

**Authors:** Yu Du, Shaochuan Fu, Changxiang Lu, Qiang Zhou, Chunfang Li

**Affiliations:** ^1^School of Economics and Management, Beijing Jiaotong University, Beijing 100044, China; ^2^Institute of Information Engineering, Chinese Academy of Sciences, Beijing100195, China; ^3^School of Cyber Security, University of Chinese Academy of Sciences, Beijing100195, China

## Abstract

This paper presents a simultaneous pickup and delivery route designing model, which considers the use of express lockers. Unlike the traditional traveling salesman problem (TSP), this model analyzes the scenario that a courier serves a neighborhood with multiple trips. Considering the locker and vehicle capacity, the total cost is constituted of back order, lost sale, and traveling time. We aim to minimize the total cost when satisfying all requests. A modified deep Q-learning network is designed to get the optimal results from our model, leveraging masked multi-head attention to select the courier paths. Our algorithm outperforms other stochastic optimization methods with better optimal solutions and *O*(*n*) computational time in evaluation processes. The experiment has shown that reinforcement learning is a better choice than traditional stochastic optimization methods, consuming less power and time during evaluation processes, which indicates that this approach fits better for large-scale data and broad deployment.

## 1. Introduction

With online retail sales rising steadily, challenges arise from the vast express delivery business volume, consumers' strong demand for convenience, and last-mile delivery. Now, consumers' expectation for delivery service is changing by hour rather than by day. The relatively higher price in physical stores is now driving more and more consumers to online shopping. Paying minor freight insurance ahead exempts consumers from shipment tariffs, provided they are not satisfied with the commodity. This kind of shopping is popular among college students and white collars who are too busy to visit the supermarkets.

However, along with safety issues, many other problems may arise in home-delivery. The proper time between customers and couriers could not be arranged. Self-pickup points and grocery stores cooperating with mail and courier companies are the main alternatives to home-delivery for online shopping delivery [1]. Nevertheless, most physical stores do not run 24 hours a day, making it hard for consumers to take away their parcels. The different size of parcels often messes up the layout of physical stores. The worst scenario is parcel loss.

To address the above issues, parcel lockers are introduced and popularized rather quickly. Walmart, Alibaba, or jd.com have recently undertaken a sustained reengineering of their distribution networks, including wide use of parcel lockers. Lockers feature easy accessibility, convenience, and safe delivering and retrieving. They are of various sizes, each with an electronically controlled release operated door latch. The lockers' smart access system functions to realize password generation and password authentication. These parcel lockers are always placed at the front gate of a neighborhood or in the parking lots. When parcels are all put in the locker, customers need to walk a distance to take them away. However, such delivery is not desirable when customers are waiting for home-delivery at home, which may breed consumer dissatisfaction and lead them to give negative reviews to online stores. More factors need to be considered to popularize the use of parcel lockers better. Some authors proposed designing a distribution network, including parcel lockers, to solve the last-mile problem [2]. Their objective considers the loss of potential customers who are not willing to walk a distance. Soliciting the recipients' willingness in advance may reduce misunderstanding and further streamline the delivery process.

Using express lockers and home-delivery simultaneously is a new strategic approach to this problem. Recipients can choose which way to receive parcels when making orders. Couriers will ask again before visiting the predetermined place. If customers still prefer lockers or give no feedback, the couriers simply head for the lockers. Once the parcel is put in the locker, the customers will be alerted on their smartphone to the message, including the locker's verification code and the due time of picking up. Customers can arrange their time and take away their parcels any time using the verification code. The express locker is 24h available. In this kind of service, the customers' need for time freedom is fully met. They are saving the waiting time of the courier, communication links, and further improving distribution efficiency. In reality, couriers also need to retrieve the parcels from customers. Some customers prefer to self-put the parcels when they pass by a locker, while only a small percentage want to make an appointment and wait for home-pickup. So, it is of great importance to solve the simultaneous pickup and delivery (SPD) problem.

SPD problem of this kind is similar to that of battery replacement in the shared electric vehicles. When the battery is exhausted or falls below a certain threshold, it is difficult for users to continue. Vehicle companies then turn to manual battery replacement for a solution. Some companies have a centralized station for battery replacement, where customers can replace the battery themselves. In this case, operators need to transport the battery to the centralized station. Otherwise, they need to replace the battery in some scattered places. Centralized battery replacement station is similar to lockers, while scattered places are like customers. Customers' parcel picking up and delivering speed at the locker is unknown, so both locker and vehicle capacity should be considered. Most scholars consider the “hard-constraint” of capacity when designing the network [3]. Suppose customers' speed putting parcels into the locker is already known. In that case, the company can arrange the delivery route in advance and consider both the locker and vehicle's real-time capacity. Overloading is nonexistent in this arrangement. However, in reality, customers are not rational enough. The speed of putting parcels into the locker and retrieving parcels is hardly predictable. When couriers arrive at a parcel locker, they may find that the lockers' capacity is not the same as the starting route. They still need to proceed to handle the excess parcels, which require space for placement. Therefore, we propose a capacity-calculating method, considering the extra amount when the production rate of resources is inconsistent between producers and consumers. Some points will have back order when couriers are undergoing a trip and then the next trip begins. When the courier visits customers' homes for home-delivery, there will be lost sale if customers want to deliver parcels but the courier cannot sustain. When the customers use an express locker, the excess number of parcels will be regarded as another lost sale.

In this new context, SPD's distribution network considering locker and vehicle capacity is taken into strategic considerations. Under this circumstance, the intelligent parcel locker can meet more needs for its high resource turnover rate. We address the simultaneous pickup and delivery problem on express lockers (SPDEL) in this paper. Pickup and delivery routing, vehicles' capacity, and lockers' use are often studied separately in the current literature. We generalize several pickup and delivery between customers and couriers using express lockers problem settings. These lockers are storage units for both customers and couriers. Only one courier is studied in our problem settings and may have several trips. The problem is known as a variant of the traveling salesman problem (TSP). We can also name SPDEL as a multitrip simultaneous pickup and delivery traveling salesman problem (MSPDTSP).

In route designing for MSPDTSP, computational complexity arises as considering capacity for lockers and vehicles. The more integrated these problems are, the more efficient a routing system can be. The problem addressed in our paper is similar to Azizi and Hu [4] and Baniasadi et al. [5]. Unlike Azizi and Hu [4], we consider SPD among lockers and customers. Our motivation is to design a distribution network that allows a courier to pickup and deliver more parcels by making fewer trips, a move aimed at lowering labor cost and boosting revenue. Different from Baniasadi et al. [5], we use a three-layer distribution network. The first layer is divided by the courier's trip routes, with one trip standing for a cluster. The courier visits every subcluster in each cluster in the middle layer, which is the express locker in our problem. The internal layer is constituted by the customers who request home-delivery. The courier visits every point in each subcluster. The customers are accessible only once, but lockers can be visited many times.

Besides the new model, we also design a new algorithm for the model's optimization. Traditionally, researchers rely on stochastic optimization methods, such as simulated annealing algorithms [6], particle swarm optimization algorithms [7], and genetic algorithms [8], to deal with complex constraints in various TSP models. Some authors put forward neighborhood search to deal with the routing problem. Wang et al. [9] proposed three kinds of operators to solve the location-routing problem with time windows and analyzed the effectiveness. Others use ant colony algorithms [10] due to the robustness and adaptation of the model. Gao et al. [11] described a clustering ant colony algorithm to tackle the dynamic location-routing problem. The utilization of K-means clustering significantly improved the performance. However, these methods are inefficient. Recently, researchers apply deep learning methods to routing issues [12–14]. Inspired by their work, we design a new reinforcement learning (RL) method for MSPDTSP. From the conventional RL's perspective, the environment in MSPDTSP continuously changes its action space in every step. As a result, we introduce the masked multihead attention mechanism from transformer [15] into the RL, hiding those inaccessible points using masks in policy networks. Our new optimization algorithm is named attention route planning network, which runs much faster than those stochastic optimization methods because our algorithm is an end-to-end system. We can train it well with generated data before deployment.

The back order and lost sale are also taken into consideration. To the best of our knowledge, the MSPDTSP has not been addressed in the previous literature. Our study's main contributions are as follows. First, our work enriches the aspect of traditional TSP to a three-layer distribution network that enables companies to deal with parcel delivery and pickup at the same time. Second, we combine the express lockers with TSPSPD and address express lockers' function as storage units for both couriers and customers. Third, we introduce back order and lost sale punishment to constrain the time for different delivery and pickup procedure. Fourth, we apply a modified RL method named attention route planning network for optimization, dealing with our model's complex constraints. Finally, we employ and compare the small, medium, and large instances and give some managerial insights that can serve as future management direction for express and mail companies.

The remainders of the paper are organized as follows.  Section 2 briefly reviews and summarizes the literature about TSP and related algorithms.  Section 3 presents the proposed MSPDTSP description and formulation. Our modified deep Q-learning network (DQN) is described in Section 4. Results of the model and numerical discussions are detailed in Section 5. Section 6 illustrates the conclusions, managerial insights, and future work.

## 2. Literature Review

### 2.1. Multitrip TSP

TSP has commanded much attention because it is easy to describe but difficult to solve [16]. The problem can simply be stated as finding the least costly route that a traveling salesman visits exactly once each of a list of *m* cities and then returns to the home city. Variations of the TSP have been studied extensively in literature [17].

In most studies, the salesman in TSP only performs one trip, i.e., single trip. However, multitrip TSP (MTSP) cannot be ignored. Babaee Tirkolaee et al. [18] studied an MTSP related to urban waste collection, a municipal activity with high costs, and many practical difficulties. Unlike the classical multitrip vehicle routing problem that several vehicles start to work together, MTSP is that one salesman travels many times in the planning horizon. Masmoudi et al. [19] integrated the multitrip concept in the Dial-a-Ride Problem, where the vehicle can perform several trips per day. Zhang et al. [20] studied a real-life public patient transportation problem. To prevent the spread of diseases, ambulances must return to the depot during the day for sterilization. Qin et al. [21] investigated an MTSP, where the salesman is permitted to return to the depot more than once. The travel cost is composed of the transportation cost and traveling allowance. Moreno et al. [22] proposed that vehicles can be reused for multitrips over periods compared with existing models. In reality, the multitrip feature is needed when the vehicles' fleet size is limited or the routes have a fixed duration.

### 2.2. TSP with Express Lockers

Some authors have studied TSP combining a truck and a drone that gives rise to a new distribution network designing problem known as the TSP with the drone [23]. Concerning truck-drone systems, researchers have proposed TSP with a drone station to solve a truck-drone system to overcome the flight-range limitation [24]. The wide use of express lockers induces the study of TSP with lockers. Enthoven et al. [25] introduced that parcels can be transported to two types of locations, namely, covering locations such as parcel lockers that customers can pickup parcels themselves and satellite locations where parcels are delivered to customers. Customers indicate their choice for delivery. The advantage of direct delivery to satellite locations is that customers can verify the commodities face to face. Its drawbacks, however, are unfixed delivery time and customers' safety. The indirect delivery requires couriers to deliver the parcel to the nearest express locker. Arnold et al. [26] analyzed the scenario that randomly chose some customers who encounter the failure of delivery, i.e., customers need to pickup their parcels from delivery points like parcel lockers. This way is compared with traditional home-delivery. And the result shows that delivery points reduce the travel time of freight. Veenstra et al. [27] introduced a location routing problem, proposed that patients within the locker's coverage distance can collect their medication, and determined which lockers need to be open. Also, it generated the opened lockers' visiting routes and the patients that are not covered by the opened lockers. Deutsch and Golany [2] considered an uncapacitated facility location problem, including a parcel locker network designed to solve the last-mile issue and calculated the loss of potential customers unwilling to use the locker. Most literature only focuses on solving the problem of using parcel lockers to deliver to the end consumers. However, the reversed problem, picking up the parcels from the consumers' locations and transferring them to DC, is not considered.

Some authors have analyzed the benefits of using express lockers with home-delivery. Pham and Lee [28] used cost-benefit analysis to study the costs and benefits of installing parcel lockers regarding the government and society's growing concern over the security issue. Then, they introduced unmanned parcel lockers as an alternative for home-delivery services. Unlike the traditional delivery model in which each customer prefers a single location to receive the parcel, service points like parcel lockers may provide several locations for recipients to choose from lowering the transportation cost and delivery time [29]. Van Duin et al. [30] also addressed in a case study that parcel lockers have a high potential to save cost. Schwerdfeger and Boysen [31] compared the mobile parcel lockers with their stationary counterparts, quantified mobile parcel lockers' positive effects, and concluded that up to 400% more lockers are required when applying stationary instead of mobile lockers. Our paper's pickup express lockers which are named collection and delivery point by Punakivi [32]. A comparative analysis of the attended and unattended reception in many aspects has demonstrated that the unattended reception can cut one third of the delivery cost compared with the former. Lockers also arouse consumers' interest in participating in packaging recycling. Cardboard boxes are directly collected through the recycling center for secondary use. Nonrecyclable packaging, such as tape and plastic bags, are collected by the environmental protection department and processed uniformly.

### 2.3. TSP with Pickup and Delivery

Express lockers in our paper serve as both recipients and providers. Couriers are picking up and delivering parcels at the same time. Hernández-Pérez et al. [33] proposed one-commodity pickup and delivery TSP in a set of customers. Each of them supplies (pickup customers) or demands (delivery customers) a given amount of a single product. It is assumed that any product collected from a pickup customer can be delivered to any delivery customer. The sequential is predetermined in Kalantari et al. [34] that each pickup customer must be visited before its associated delivery customer. Once a demand has been picked up, it can only be dropped off at its desired delivery location [35]. TSP with pickup and delivery (TSPPD) is a challenging variant of TSP that includes the transportation of commodities between locations. Castro et al. [36] proposed that TSPPD is to find a minimum cost trip such that each item is delivered to its destination, and the capacity of the vehicle is not exceeded. In our paper, each point in the customers' set may have two demands. One requires several parcels (delivery demand); the other provides several parcels (pickup demand). The parcels to be delivered are originated from the distribution center. Couriers can simultaneously pickup and deliver parcels based on the customers' needs.

### 2.4. Deep Learning Methods

Heuristics have effectively solved the standard VRPSPD instances of Salhi and Nagy [37]. Kóczy et al. [38] developed a novel metaheuristic named discrete bacterial memetic evolutionary algorithm to solve TSP. The results show that it was faster than the Concorde solver in large size instances but slower than the most efficient TSP solver method Helsgaun-Lin-Kernighan heuristic. Zhong et al. [39] proposed a hybrid discrete artificial bee colony algorithm that can learn from other bees and features of the problem at hand. The algorithm is competitive with many different state-of-the-art algorithms. Hussain et al. [40] used genetic algorithms and proposed a new crossover operator for TSP to minimize the total distance.

However, these stochastic optimization algorithms require a lot of time and computing power to deal with different circumstances every time. In contrast, it is unacceptable for courier companies to deploy enough equipment in each distribution center as it costs too much. As a result, we apply an end-to-end RL method to get the optimized result. After the training process, we can deploy this kind of optimization method with its pretrained model on regular personal terminals, such as PC and mobile phones.

In recent years, researchers apply various end-to-end neural networks to routing problems. Kaempfer and Wolf [41] introduced a learning-based TSP solver, designing the permutation invariant pooling network with the residual mechanism and normalization layers. Nazari et al. [42] presented an end-to-end framework as a VRP solver, combining the RL model with the original attention mechanism. Kool et al. [43] applied the multihead attention model to deal with routing problems which broadens the solver's scope to both TSP and VRP problems since the multihead attention model can focus on different features in different environments.

In summary, our paper considered the multiple trips for traditional TSP regarding couriers which usually serve a fixed area many times in a day. We enriched the study of TSP with express lockers by considering the home-delivery together. Unlike most papers that consider pickup or delivery, we address SPD because some customers may return parcels in reality. This MTSP with simultaneous pickup and delivery on express lockers has not yet been studied in previously integrated contexts. Specifically, we are interested in a three-stage distribution network that considers the capacity of express lockers and vehicles. Moreover, we introduce back order and lost sale calculation when the parcels are in a different state. To solve the model, we apply a modified reinforcement learning method. Our problem is described as a multitrip, simultaneous pickup and delivery, and traveling salesman problem with express lockers.

## 3. Problem Statement and Model

Our paper addresses a distribution route designing problem considering simultaneous pickup and delivery involving distribution center (DC), express lockers (EL), and customers. The courier starts from DC carrying parcels to deliver, visiting lockers, and a set of customers. The courier then returns to DC. We assume that there is one DC in the area, and several lockers are placed in advance. EL can serve customers living in the area, but it is up to the customers to decide whether to use EL or not. If not, the customer can request home-delivery. Each EL and customer point includes three attributes, coordinates, the number of parcels required (delivery demand), and the number of parcels provided (pickup demand). The following picture shows an example of considering using EL. A part of customers request home-delivery (e.g., C7 and C8), while most of them (e.g., C1 and C2) prefer using EL.

**Figure 1:**
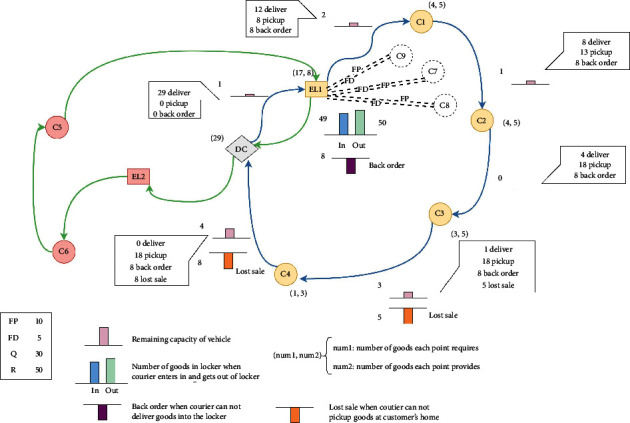
Route considering using EL.

The problem is depicted in Figure 1. A courier starts from DC (blue line) and then goes to the first express locker (EL1). In this neighborhood, 4 customers (C1, C2, C3, and C4) need home-delivery and have already been designed in route. However, EL has a random speed of retrieving (FP) and receiving (FD) parcels from other customers (C7, C8, and C9). When the courier starts, the vehicle loads 29 parcels, and the remaining capacity of the vehicle is 1. EL1 has a required amount of 17 and provides 8. Owing to the uncontrolled speed (FP and FD), EL's capacity is only 1 when the courier arrives. The courier puts 9 parcels in EL1, considering both FP and FD. The rest 8 parcels are back orders waiting for another visit to satisfy. The remaining capacity of the vehicle is 2. When the courier goes to the first customer (C1) designed to use the locker in EL1's serving area, the customer requires 4 and provides 5. After exchanging the same number of parcels, 1 exceeded delivering parcel will be picked up by the courier and the remaining capacity of vehicle minus 1.

Though C3 was predetermined in the route, the vehicle's remaining capacity cannot satisfy the number of parcels C3 provides. Customers' demand cannot be split since customers do not want to be visited many times. The whole 5 parcels are lost sales, and C4 is the same. The courier returns to DC with all parcels delivered, 18 parcels picked up, 8 back orders, and 8 lost sales. Then, the next route is ready, and 8 back orders of EL1 will be redesigned this time (green line). When all delivery and pickup demands of the day are made, the courier's task is completed. The back order amount can be redelivered, but the lost sales are lost.

To better understand how to calculate back order and lost sale, we introduce the state of parcels to describe different phases of delivering and picking up parcels. From the customers' view, when they decide to deliver parcels to the locker, the parcels are *On the Way*, the first state. When they compare the number of parcels with the locker's remaining capacity, the parcels are *Comparing*, which is the second state. When they finish putting parcels into the locker, the parcels are *In Place*, the third state. The lost sale takes place in the second state. Three states are *On the Way*, *Comparing*, and *In Place* when visiting each point from the courier's view. When the courier serves EL, a back order could take place in the second state. When the courier serves customers, the lost sale could take place in the second state.

**Figure 2:**
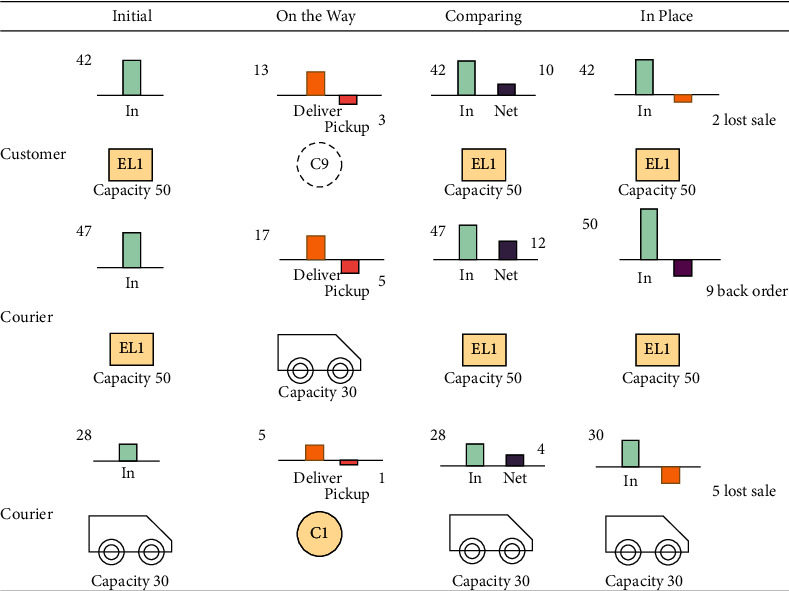
Three states of the parcel.

Figure 2 shows examples of three states of parcels. When customers visit EL1, the initial number of parcels in EL1 is 42, and the locker's capacity is 50. In the first state, i.e., *On the Way* state, the customers provide 13 parcels and require 3. In the second state, i.e., *Comparing*, parcels' net demand is 10, which means 10 parcels need to be put into the EL1. However, EL1 can only sustain 8. The rest 2 parcels are regarded as lost sales. The remaining capacity and lost sale are updated in the *In-Place* state. When a courier arrives at EL1, the initial capacity of EL1 is 47. The courier is carrying 17 parcels in the *On the Way* state and ready to pickup 5 parcels. After calculating parcels' net demand in the *Comparing* state, 9 parcels are left as back orders, waiting for another delivery trip. The remaining capacity and back order are updated in the *In-Place* state. When a courier arrives at a customer's home, the lost sale occurs in the *Comparing* state. Customers' demand cannot be split when they request home-delivery.

### 3.1. Model Assumptions

In this section, we first define the problem in general terms. We start by describing the basic assumptions. Afterward, the parameters and decision variables are included. Then, the constraints are presented. Our goal is to find a reasonable route that minimizes the total set up, back order, lost sale, and transportation cost.

The basic assumptions for our model are as follows:

- Each trip should start from and return to DC. The vehicle has unlimited driving distance, and its capacity is not exceeded.
- Trip order is to go to EL in this neighborhood and then to the rest of the customers who request home-delivery. The courier then moves to the next community. If there is no request for home-delivery in the next neighborhood, the courier can go from this EL to the next EL.
- Every customer can and only can be served once, while EL can be visited multiple times but only once in a single trip. The traffic situation is not considered in our paper.
- Unsatisfied demands are either classified as back order or lost sale. If the locker is full when the courier has new parcels to deposit, the exceeded number will be identified as a back order. Therefore, there is a time cost, and the back order is waiting for the next trip to serve. If the customers' parcels go beyond the vehicle's capacity, the whole amount is deemed to be lost sale. If the customers' parcels go beyond the locker's capacity, the exceeded amount is considered as lost sale.

### 3.2. Notation

Table 1 shows the indices, sets, parameters, and decision variables included in our formulation.

### 3.3. Model Formulation

As shown from the above discussion, the Without Express Lockers Scenario is a special case of Using Express Lockers Scenario. In the Without Express Lockers Scenario, the courier starts from DC and then visits a set of customers based on the demand origins from DC and picks up parcels from customers. The objective function can be expressed as follows:

(1) $$\begin{array}{c} \begin{array}{c} \min z_{1}=\sum_{k\in K}\sum_{i\in P\cup^{}O}\sum_{j\in P\cup^{}O}T_{ij}x_{ij}^{k} \end{array}. \end{array}$$

The goal is to minimize the total transport time between every two points in the planning horizon. The following constraints formulate the detailed process of this scenario:

(2) $$\begin{array}{c} \sum_{j\in P\cup^{}O}\sum_{k\in K}x_{ij}^{k}=\sum_{j\in P\cup^{}O}\sum_{k\in K}x_{ji}^{k}=1,\;\forall i\in P\cup^{}O, \end{array}$$

(3) $$\begin{array}{c} \sum_{j\in P\cup^{}O}x_{ji}^{k}=\sum_{j\in P\cup^{}O}x_{ij}^{k},\;\forall i\in P\cup^{}O,\;k\in K, \end{array}$$

(4) $$\begin{array}{c} \sum_{i,j\in P^{\prime }}x_{ji}^{k}\leq \left|P^{\prime }\right|-1,\;\forall P^{\prime }\subseteq P\cup^{}O,\;\left|P^{\prime }\right|>1, \end{array}$$

(5) $$\begin{array}{c} x_{ii}^{k}=0,\;\forall k\in K,\;i\in P\cup^{}O. \end{array}$$

Constraints (2) and (3) verify that each node is visited at most once, and Constraints (4) and (5) are defined to forbid the formation of subtours.

(6) $$\begin{array}{c} I_{o}^{k}=\sum_{i\in P}d_{i}^{k},\;\forall k\in K,\;o\in O, \end{array}$$

(7) $$\begin{array}{c} I_{j}^{k}=I_{j-1}^{k}+p_{j-1}^{k}-d_{j-1}^{k},\;\;\forall j\in P\cup^{}O,\;k\in K, \end{array}$$

(8) $$\begin{array}{c} I_{j}^{k}\leq Q,\;k\in K,\;\forall j\in P\cup^{}O. \end{array}$$

Constraints (6)–(8) model the vehicle capacity for each trip. In the beginning, the courier loads the parcels for a single trip and starts from DC. In each trip, the vehicle load is equal to the load when the courier arrives at the former point plus the pickup demand and minus delivery demand of the former point.

In the Using Express Lockers Scenario, customers are assigned to lockers. The courier serves the locker and then goes to the customers' sets which belong to this locker but request home-delivery. If the locker is exceeded, there will be a back order. Also, we introduce two kinds of lost sales in which a customer wants to deliver, but the vehicle or locker capacity cannot sustain.

The courier must finish all the delivery and pick up demand of the day. Owing to the capacity of the vehicle, they may make multiple visits. Minimizing the number of trips is one way to lower the operation cost of setting up.

(9) $$\begin{array}{c} z_{2}=\sum_{k\in K}\sum_{u\in U}x_{ou}^{k},\;\forall o\in O. \end{array}$$

Transport time reflects the total travel distance of a courier, which may serve as a metric of moving cost or the workload.

(10) $$\begin{array}{c} z_{3}^{k}=\sum_{i\in V}\underset{j\in V}{\sum }T_{ij}x_{ij}^{k},\;\forall k\in K,\\ z_{3}=\sum_{k\in K}z_{3}^{k}. \end{array}$$

Lost sale when the customers want to deliver at locker but fails is calculated as follows and represents customers satisfaction:

(11) $$\begin{array}{c} z_{4}=\sum_{k\in K}\sum_{u\in U}CU_{u}^{k}. \end{array}$$

Another lost sale when the customers want to deliver at home but fails is also a metric of customers' satisfaction.

(12) $$\begin{array}{c} z_{5}=\sum_{k\in K}\sum_{i\in N}HU_{i}^{k}. \end{array}$$

Back order occurs when the courier delivers parcels to the locker, but the locker is full. During every trip, the back order will be calculated until the courier finishes the delivery.

(13) $$\begin{array}{c} z_{6}=\sum_{k\in K}\sum_{u\in U}RU_{u}^{k}\left(z_{3}^{k}-\left(t_{u}^{k}-t_{o}^{k}\right)\right),\;\forall o\in O. \end{array}$$

The fixed setting up cost of starting is *C*_2_ per trip. Transportation cost between the network's nodes corresponding to traveling time is *C*_3_ per min. Lost sale punishment is *C*_4_ per parcel when the customers use express lockers *C*_5_ per parcel when they request home-delivery. Back order punishment is *C*_6_ per parcel when courier uses express lockers to deliver. To minimize the total cost, we can integrate the five costs into one.

(14) $$\begin{array}{c} \begin{array}{c} \min z=\left(C_{2}z_{2}+C_{3}z_{3}+C_{4}z_{4}+C_{5}z_{5}+C_{6}z_{6}\right) \end{array}, \end{array}$$

s.t.

(15) $$\begin{array}{c} \sum_{u\in U}x_{ou}^{k}=1,\;\forall k\in K,\;o\in O, \end{array}$$

(16) $$\begin{array}{c} \sum_{j\in V}x_{ji}^{k}=\sum_{j\in V}x_{ij}^{k}\;,\;\;\forall i\in V,\;k\in K, \end{array}$$

(17) $$\begin{array}{c} x_{ii}^{k}=0,\;\forall k\in K,\;i\in V, \end{array}$$

(18) $$\begin{array}{c} \sum_{j\in V}\sum_{k\in K}x_{ij}^{k}=\sum_{j\in V}\sum_{k\in K}x_{ji}^{k}=1,\;\forall i\in N, \end{array}$$

(19) $$\begin{array}{c} \sum_{i,j\in V^{\prime }}x_{ji}^{k}\leq \left|V^{\prime }\right|,\;\forall V^{\prime }\subseteq V,\;\left|V^{\prime }\right|>1. \end{array}$$

Constraint (15) shows that EL is to be visited first in each trip. Constraints (16)–(19) express that each node is visited only once in a single trip and forbids subtour. Constraint (18) is mainly for customers who request home-delivery because EL can be visited multiple times in the planning horizon. However, customers can be visited only once.

(20) $$\begin{array}{c} I_{o}^{k}=\sum_{i\in V/O}d_{i}^{k},\;\forall k\in K,\;o\in O, \end{array}$$

(21) $$\begin{array}{c} I_{j}^{k}=\left(I_{j-1}^{k}+p_{j-1}^{k}-d_{j-1}^{k}\right),\;\forall j\in V\backslash O,\;k\in K, \end{array}$$

(22) $$\begin{array}{c} I_{j}^{k}\leq Q,\;\forall j\in V,\;k\in K. \end{array}$$

Constraints (20)–(22) model the vehicle capacity for each trip. The vehicle load is equal to the load when the courier arrives at the former point plus the pickup demand and minus delivery demand.

(23) $$\begin{array}{c} cd_{u,2}^{k}=\left(R-h_{u,2}^{k}\right)+\text{FP}\left(t_{u}^{k}-t_{u}^{k-1}\right),\;\forall k\in K,\;u\in U, \end{array}$$

(24) $$\begin{array}{c} cd_{u,3}^{k}=\min\left\{\text{FD}\left(t_{u}^{k}-t_{u}^{k-1}\right),cd_{u,2}^{k}\right\},\;\forall k\in K,\;u\in U, \end{array}$$

(25) $$\begin{array}{c} CU_{u}^{0}=0,\;\forall u\in U, \end{array}$$

(26) $$\begin{array}{c} CU_{u}^{k}=\max\left\{\text{FD}\left(t_{u}^{k}-t_{u}^{k-1}\right)-cd_{u,3}^{k},0\right\},\;\forall k\in K,\;u\in U. \end{array}$$

Our paper's difference is that MSPDTSP considers delivering parcels to customers and picking up parcels that customers provide at EL. In reality, the locker's capacity is not unlimited. If customers want to put parcels in while the locker is full, lost sale punishment can be used to calculate the cost of customers' dissatisfaction. Equations (23)–(26) depict the procedure customers put parcels into EL. Consider that FP and FD obey a certain distribution. EL may be full when customers arrive. If the number of parcels EL can receive is lower than that of parcel customers wants to put in, then the difference between the two numbers is customers lost sale *CU*_*u*_^*k*^ and equals to zero at first.

The courier starts from DC and first goes to EL. If no customer wants to receive parcels at home in this area, the courier can move between lockers or return to the DC. It is not allowed to return to the previous locker provided that a courier finishes putting the parcels into the locker and moves on to the next customer in the same area. Considering most ELs are at the neighborhood's front gate, we assume that the courier can choose the shortest path during work.

(27) $$\begin{array}{c} h_{u,1}^{k}=h_{u,3}^{k-1}-\text{FP}\left(t_{u}^{k}-t_{u}^{k-1}\right)+cd_{u,3}^{k-1},\;\forall k\in K,\;u\in U, \end{array}$$

(28) $$\begin{array}{c} h_{u,3}^{k}=h_{u,1}^{k}-p_{u}^{k}+d_{u}^{k},\;\forall k\in K,\;u\in U, \end{array}$$

(29) $$\begin{array}{c} h_{u,1}^{k},h_{u,3}^{k}\leq R,\;\forall k\in K,\;u\in U. \end{array}$$

Equations (27)–(29) are express locker constraints, and *h*_*u*,1_^*k*^ and *h*_*u*,3_^*k*^ represent different phases when the courier arrives at EL. When the courier just arrives, the number of parcels EL can sustain is calculated by *h*_*u*,1_^*k*^. When the courier leaves, it is recalculated by *h*_*u*,3_^*k*^ considering the number of delivering and picking up at the EL.

(30) $$\begin{array}{c} rp_{i,2}^{k}\;=Q-I_{i-1}^{k}+d_{i}^{k},\;\forall i\in N\cup^{}U,\;k\in K, \end{array}$$

(31) $$\begin{array}{c} rp_{i,1}^{k}=\left\{\begin{array}{cc} \left(rp_{i,1}^{k-1}-p_{i}^{k-1}\right)+cd_{i,3}^{k}, & \forall k\in K,\;i\in U,\\ p_{i}^{k}, & \forall k\in K,\;i\in N, \end{array}\right) \end{array}$$

(32) $$\begin{array}{c} p_{i}^{k}=\min\left\{rp_{i,2}^{k},rp_{i,1}^{k}\right\},\;\forall i\in P\cup^{}U,\;k\in K, \end{array}$$

(33) $$\begin{array}{c} HU_{i}^{k}=\left(rp_{i,1}^{k}-p_{i}^{k}\right),\;\forall i\in U,\;k\in K, \end{array}$$

(34) $$\begin{array}{c} HU_{i}^{k}=\left\{\begin{array}{cc} rp_{i,1}^{k}, & rp_{i,1}^{k}>rp_{i,2}^{k},\\ 0, & rp_{i,1}^{k}\leq rp_{i,2}^{k}, \end{array}\right)\;\forall i\in N,\;k\in K. \end{array}$$

When the courier picks up the parcels from the locker that the customers provide, there are also constraints on the pickup number. Equations (30)–(34) depict the procedure a courier visits each point. If the courier visits EL and cannot pickup all parcels, the rest will be redesigned, and transportation cost substitutes the punishment. However, if it is a customer point, customers dislike being visited multiple times. The lost sale punishment should considered the result of customers' dissatisfaction. *rp*_*u*,2_^*k*^ is the number couriers can pickup and compares with *rp*_*u*,1_^*k*^ which means the number they need to pickup. The actual number courier picks up and lost sale *HU*_*i*_^*k*^ is calculated in Constraints (32)–(34).

(35) $$\begin{array}{c} d_{i}^{k}=rd_{i,1}^{k},\;\forall i\in N,\;k\in K, \end{array}$$

(36) $$\begin{array}{c} d_{u}^{k}=\text{min}\left\{rd_{u,1}^{k},R-\;h_{u,1}^{k}+p_{u}^{k}\right\},\;\forall k\in K,\;u\in U, \end{array}$$

(37) $$\begin{array}{c} RU_{u}^{k}=rd_{u,1}^{k}-d_{u}^{k},\;\forall k\in K,\;u\in U, \end{array}$$

(38) $$\begin{array}{c} x_{ij}^{k}\in \left\{0,1\right\},\;\forall k\in K,\;i\in V,\;j\in V, \end{array}$$

(39) $$\begin{array}{c} cd_{u,s}^{k},rp_{i,s}^{k},rd_{i,s}^{k},CU_{u}^{k},RU_{u}^{k},HU_{i}^{k},I_{i}^{k},h_{u,s}^{k},t_{i}^{k}\geq 0. \end{array}$$

Constraints (35) and (36) calculate the parcels' actual number that the courier delivers to the customer's home and EL. When the courier arrives at customers' homes, the customers can receive all parcels. However, EL may be full, and the extra parcels could become back order. Constraint (37) is back order punishment at EL, which means the courier fails to deliver the parcels when visiting EL because of its capacity. Constraint (38) describes the binary restriction of decision variables.

### 3.4. Model Analysis

We have the following propositions, which show the relationship between our MSPDTSP model and traditional TSP.

**Proposition 1 .**

MSPDTSP is the combination of GTSP (generalized traveling salesman problem) and CTSP (clustered traveling salesman problem), and it is also a variant of CGTSP (clustered generalized traveling salesman problem).

**Figure 3:**
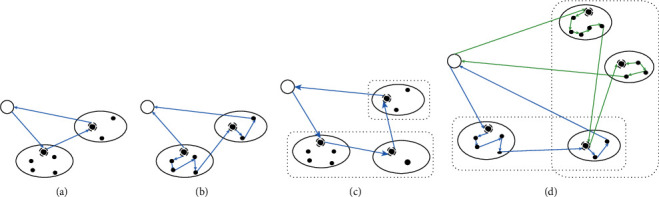
An example of (a) GTSP, (b) CTSP, (c) CGTSP, and (d) SPDEL.

According to Baniasadi et al. [5], GTSP is to visit each cluster once and find a minimum length trip that includes precisely one node from each cluster (Figure 3(a)), whereas the CTSP must visit every node in each cluster (Figure 3(b)). CGTSP is a two-layer expansion of traditional TSP in which the external layer of the problem is CTSP and the internal is GTSP (Figure 3(c)). Furthermore, MSPDTSP is a three-layer expansion of CGTSP in which the external layer is CGTSP, and the internal layer is CTSP. That is to say, upon visiting a cluster, we must visit each subcluster contained therein before moving to a new cluster. The external layer is divided by the courier's trip routes, with one trip standing for a cluster. The courier visits every subcluster in each cluster in the middle layer, which is the EL in our problem. The internal layer is constituted by the customers who choose home-delivery. The courier visits every point in each subcluster. MSPDTSP combines the features of both GTSP and CTSP and is a variant of CGTSP (Figure 3(d)).

**Proposition 2 .**

TSP is a special case of the MSPDTSP model when cutting down the locker settings.

By Proposition 1, CTSP and GTSP are special cases for CGTSP. CGTSP is when there is only one subcluster in each cluster of GTSP and is when there is only one node in each subcluster of CTSP. It is easy to see that CTSP is a TSP with restrictions. Likewise, CGTSP is a GTSP with restrictions. Moreover, the traditional TSP and MSPDTSP have identical backgrounds. Given a set of n cities and a distance matrix, the goal is to find a minimum length trip, that is to say, to start in some cities, visit each other city once, and come back to the initial city. There is only one trip in TSP that connects all the cities. However, there are multiple trips in MSPDTSP. The number of trips is constrained by the capacity of the vehicle and lockers. Moreover, lockers in MSPDTSP can be visited more than once on the planning horizon but only once in a single trip.

A Problem A is said to be a special case of problem B if an algorithm solving problem B can also solve problem A. If both vehicle and locker capacity is maximum, only one trip can satisfy all the demands, which is the same with TSP. Therefore, TSP is a special case of MSPDTSP. TSP is the occasion when the capacity limitation of MSPDTSP is infinite. MSPDTSP is as least as hard as the 0-1 integer programming problem, which proves it is NP-hard. It is widely suspected that there does not exist any polynomial algorithm for MSPDTSP. A suitable heuristic algorithm is required to obtain an approximate solution to the problem quickly.

## 4. Attention Route Planning Network

In traditional TSP problems, the environment settings are static. Each customer has their locations and requirements. The courier only needs to satisfy these requirements, sending packages from the delivery center to customers' points. However, the MSPDTSP is a dynamic programming problem in which some environment parameters vary at different moments, including inventories of lockers and the courier's vehicle. Besides, our model contains 20 constraints corresponding to the dynamic situation with ten variables, which need an extremely high-dimensional state transition matrix to present all potential routes for courier. It is low efficient to complete this matrix or search for the best path in it. Furthermore, a large-scale MSPDTSP problem will lead to computational complexity explosion for traditional heuristic algorithms, such as the L-K heuristic or ant colony algorithm. As a result, we introduce reinforcement learning (RL) for searching for an optimal course for the courier in our model.

**Figure 4:**
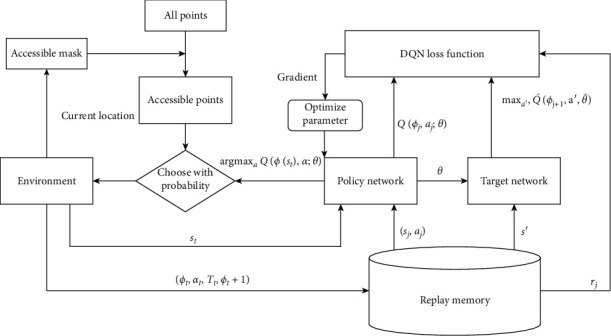
The adapted DQN framework.

RL is also called approximate dynamic programming, which is good at dealing with complex sequential choices problems. It only needs initial status, step changes, and termination conditions from environments to make an optimal decision. An RL framework consists of three modules such as an environment, an optimizer, and an action network as shown in Figure 4.

To solve the MSPDTSP problem, we first design a new environment to simulate the total circumstance that couriers need to deal with, calculating all state variables for subsequent decisions. After that, we decided to choose deep Q-learning (DQN) as the optimizer since it can handle discrete environments well. We also add some new mechanisms into the DQN's process, making it meet the MSPDTSP's requirements. Finally, we design a new action network, introducing some new techniques for a better routing strategy.

### 4.1. Environment

Unlike traditional RL settings like the gym library, the environment in MSPDTSP varies with every action because we integrate the constraints of our model into the environment model. Firstly, we initialize all discrete variables as 0 since the courier has not decided which customers or lockers to visit.

Secondly, in each step, a courier should choose a path based on the constraints. Specifically, the 20st, 21nd, and 22rd constraints claim that the vehicle's capacity must be enough for the selected destination's delivery and pickup request during the planning process. Otherwise, the courier has to choose another point. For instance, a courier at a locker point can only pick customer points in the region serviced by the locker point unless no delivery demand in this region. In this process, we introduce a masking mechanism to our environment design, conducting the courier to choose accessible destinations.

We also simulate the situations of lockers' inventory. In reality, we cannot predict when and how much postal matter could be delivered to or picked up from any lockers. Thus, we leverage the Poisson process to simulate customers' delivering and picking up packages to calculate lockers' inventory in each step, following equations (23)–(29). Since lockers' inventory relates to random processes, we should consider that the courier could not complete the mission. Hence, we need to recalculate how many packages are carried by courier from a point as Constraints (30)–(32). Meanwhile, the courier may fail to put some parcels into lockers when the lockers' capacity is lower than our expectation. Consequently, the courier fails to deliver all packages to those lockers as previous plan. So, we have to know the actual delivery number based on the 36th and 37th constraints. Besides, we need to log various costs during each step, including lockers' lost sales (26), courier' lost sales (33) and (34), and back orders (37).

At last, the termination condition is to accomplish all delivery missions. After that, we will accumulate all kinds of costs for each tour, which will be used to optimize the routing selection strategy model.

### 4.2. Optimizer

In this paper, we leverage DQN as the optimizer. The DQN is a simple but suitable method for our problem since the MSPDTSP's action space is discrete with limited sizes. However, some details in the DQN's framework are not fit for the MSPDTSP problem. The first one is the sequence ending condition. As our model has an explicit termination condition, we remove the termination turns hyperparameter and log the number of turns to match other parts of the DQN framework at line 6 and line 23 in Algorithm 1. The other contrast shows at line 9 in Algorithm 1 that our action space changes in different situations, which requires us to mask those illegal points to guide the courier in selecting a path in an accessible range.

### 4.3. Action Network

The most effective part of our algorithm is the action network. Based on the settings of MSPDTSP, a courier chooses the path mainly depending on three factors, including distances, delivery demands, and pickup demands. Regularly, the best path choice is the shortest one. Nevertheless, the courier also needs to pay attention to the express car's inventory related to delivery and pickup demands. As a result, we combine these three factors to extract nodes' embedding vectors. We set its three-dimensional feature as the input and leverage a fully connected neural network, a simplified version node2vec model, as the embedding network to generate a 32-dimensional embedding for each node.

We generate node embeddings before each step. After that, we need to invalidate unreachable points following the mask from the model. Here, we use the multihead attention model from the transformer. The attention mechanism forces the model to pay more attention to nodes with higher weights. The multihead attention further introduces masks, allowing programmers to directly hide some features, which meets the MSPDTSP's masking mechanism requirement.

Moreover, the multihead attention model combines multiple factors with weight matrices, similar to the different MSPDTSP problem circumstances. Therefore, we feed embedding vectors from the fully connected network and the mask from the MSPDTSP model to our multihead attention model to choose the best way for the courier. Specially, we split the last action's embedding vector as the query, using all embeddings from the embedding network as keys and values. Besides, we reshape the access mask from the MSPDTSP environment to fit the multihead attention's model.

## 5. Results and Discussion

This section presents numerical experiments to compare the small, medium, and large size instances solved by reinforcement learning methods. We examine the effect of the instance size of flexibility on the model.

We implement our model and RL method with PyTorch 1.7.1 based on Python 3.6.8. The experiments were performed on a system with Intel Xeon Silver 4214 2.2 GHz CPUs (48 processors), 128 GB memory, and Nvidia Tesla V100 GPUs with 16 GB display RAM running CentOS 7.3. We generated 10,000 instances for three sizes of configurations, with different point's coordinates and demands. The values of parameters for different sizes of instances are given in Table 2. We evaluate our model and optimization method with ten instances for each configuration as specified below.

Each instance has a homogeneous courier with the vehicle's capacity of 50 units in small size instances and 80 in medium and large size. The locker's capacity is 80 units in small size and 100 in medium and large sizes. Customers' speed picking up and delivering parcels at the locker are consistent between medium and large sizes. However, the courier serves more lockers in the large size than the medium one, which generates more points for the algorithm to calculate. The delivery and pickup demands originate from DC which are the same in each size. The pickup demand could be zero owing to the customer has no parcels to deliver. During training processes, we use FP and FD expectations as fixed speeds to improve the training stability of our modified DQN. In contrast, evaluation requires a more realistic environment. As a result, we apply a primary arrive model to FP and FD, setting their expectations as the lambdas of two Poisson distributions, respectively. We generate two random numbers lists from these Poisson distributions to resemble the number of parcels delivered to or picked up from one locker in each unit time interval.

**Figure 5:**
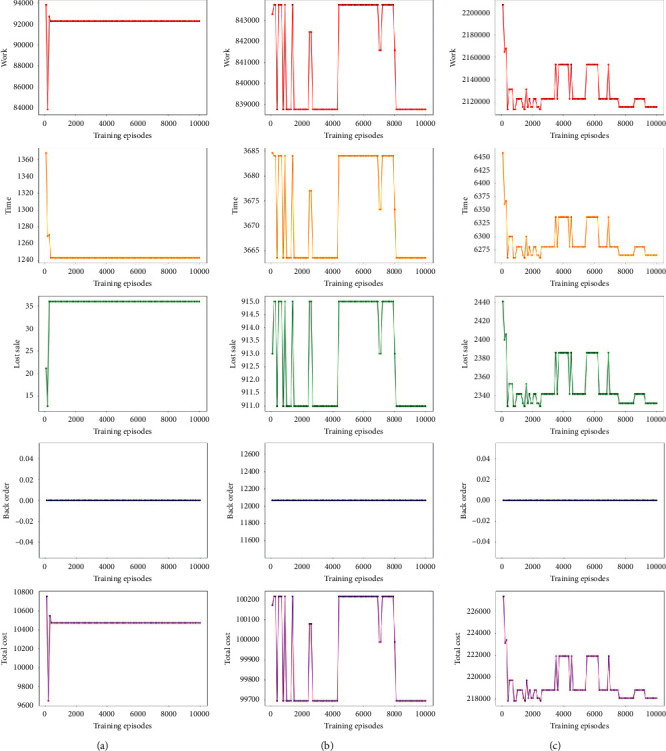
Comparison of computational results for (a) small, (b) medium, and (c) large size instances.

Two different analyses are provided. Firstly, result comparisons between the small, medium, and large instances are presented in Table 3. Secondly, back order, lost sale, time, total cost, and work cost are compared among three kinds of size instances in Figure 5.

Table 3 shows the computational results and comparisons between small, medium, and large size instances. Several important observations can be made. Firstly, small size instances have no back order. The reason is that there are only a few customers in the problem settings so that the locker can satisfy the demand of customers. Secondly, there are some back orders in the medium-sized instances and more back orders in large-sized instances. The back orders in the large size are an order of magnitude larger than those in the medium. On the one hand, the more lockers to be served under the same circumstances, the more back orders could generate. On the other hand, back order is another kind of work that needs courier to do. The whole length of large size instances is longer than that of the medium-sized which could be another reason for the large amounts of back orders. Thirdly, there are lost sales in three kinds of instances, which means the capacity of express lockers and vehicle is not sufficient. Fourthly, comparing to the small-sized, the work of medium and large size instances is an order of magnitude larger. However, the increase in work between the large and medium instances is not so massive. The computational time of medium-sized decreases 5.43% than small-sized. The large-sized increases 9.66% than the medium instances. Therefore, our algorithm's complexity is almost equal to *O*(*n*) if the delivery and pickup demands for one courier are within a reasonable interval. Our algorithm performs better on the large-sized problem in reality than conventional stochastic optimization algorithms [44].

**Figure 6:**
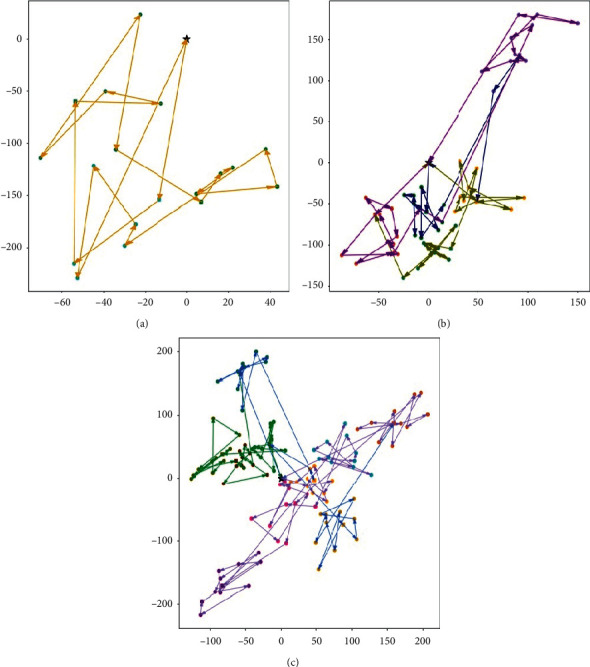
Numerical results for (a) small, (b) medium, and (c) large size instances.

Figure 6 shows three optimal routes of the test instance, respectively. DC locates at (0, 0), and each color represents a neighborhood. The multiple trips of the courier are also shown in different colors. The number of trips in medium and large size instances is more than the small-sized. It makes sense because the courier served more lockers and customers.

Figure 5 shows that the increasing number of training turns affects the test instances' results on back order, lost sale, time, total cost, and work cost. To obtain a stable performance, we generate 10,000 training turns. There is no back order in the small (Figure 5(a)(a4)) and large (Figure 5(b)(b4)) size instances. However, in the medium-sized (Figure 5(c)(c4)), the back order is not equal to zero. This is because, in the small-sized, our algorithm could solve well with the number of points. Our goal is to minimize total cost. However, the instability from DQN leads to our failure to eliminating back order in the medium-size test set. Also, the evaluation results prove our algorithm suits well in the large size instances. Furthermore, if we do not add back order cost into the cost, we could not train the model well. Then, back order will show up in the large-sized.

The results of lost sale, time, total cost, and work cost of medium and large size instances (e.g., Figures 5(b)(b3) and 5(c)(c3)) show that the metrics are fluctuating and unstable at first; after training several turns, the routing deep Q-learning performs relatively stable on these metrics. Figures 5(a)(a3) and 5(a)(a2) depict that our algorithm can rapidly optimize the lost sale in small-sized and save the picking up and delivering time.

**Figure 7:**
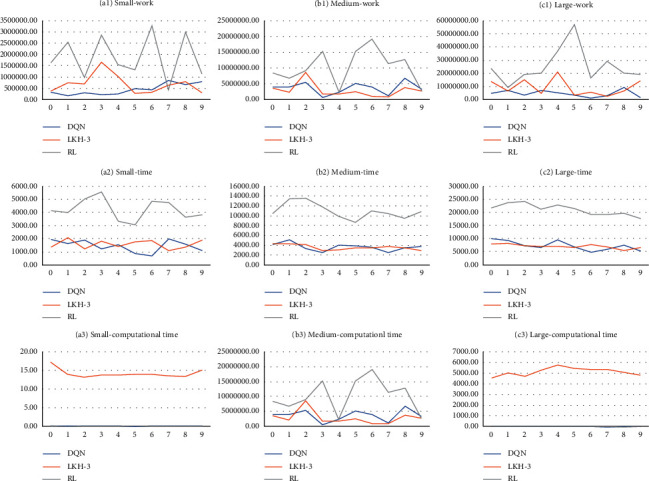
Comparison of computational results for different size instances of DQN, LKH-3, and RL.

As a consequence of previous discussions, we compare our DQN with the state-of-the-art heuristic and the traditional RL method, Monte Carlo Method [45] in Figure 7. LKH-3 [46] is a recent extension of LKH, which can be used in TSP.

Table 4 shows that the proposed adapted DQN results are promising in the experiments. Specifically, though there is no absolute advantage in work and time comparing to the LKH-3 method, the computational time saves a lot in ours. One primary reason is that corresponding to the state-of-the-art, we use the DRL method with a pretrained strategy model. The computational complexity is *O*(*n*) in contrast to *n*^2^*d*(*n*) (*d*(*n*) is the depth of the search in the algorithm) in the state-of-the-art [47]. Comparing with the Monte Carlo method, our algorithm obtained better results at work and time with the randomly generated data in each size instance. The reason is that the traditional RL is a polynomial of which the strategy function is trivial. Compared with the deep learning model, the value function's complexity is low, making it challenging to represent the high-dimensional vector space the strategy needs. Overall, the adapted DQN algorithm can simultaneously obtain a better optimal result and a low computational complexity.

## 6. Conclusion and Managerial Insight

This paper presents a new simultaneous pickup and delivery route designing model, which considers the use of express lockers. Unlike the traditional TSP, this model analyzes the scenario that a courier serves a neighborhood with multiple trips. A mathematical formulation has been developed and proved that TSP is a special case of the MSPDTSP. We apply a modified deep Q-learning network with a masking mechanism to the MSPDTSP model to obtain explainable optimal results. The policy network is an end-to-end network consisting of an embedding network and a masked multihead attention model, following the mask mechanism from the MSPDTSP's requirements. As a result, a pretrained network can deal with the MSPDTSP's complicated environments within *O*(*n*) computational time. Moreover, the pretraining helps the network perform better than other stochastic optimization methods. Numerical analyses are conducted for three different sizes of instances to evaluate the performance of the model. The first part of computational results shows that the algorithm's complexity is almost equal to *O*(*n*) if the delivery and pickup demands for one courier are within a reasonable interval. Our algorithm could well solve the small-sized problem and perform better on the large-sized problem in reality than conventional stochastic optimization algorithms. The second part of the experiments provides a comparison among five metrics. These experiments reveal that it is essential to consider the back order cost. Our algorithm rapidly optimizes the lost sale and save the picking up and delivering time. The third part of the experiments compares our algorithm with the state-of-the-art and Monte Carlo method. Overall, the adapted DQN algorithm can simultaneously obtain a better optimal result and a low computational complexity.

Considering real-life cases, the express and mail company may increase the capacity of express lockers to cut down the back order of parcels, which makes the courier service fewer trips and, in the end, improves and increases customer satisfaction. On the other hand, the vehicle's capacity is increased so that the courier can take more parcels at one time and minimize the lost sale. The company could use trucks to deliver parcels or move the location of distribution center closer to the end customers. Furthermore, increasing the turnover rate of express lockers is another way to eliminate the lost sale.

For future studies, mobile parcel lockers will indeed prove as an alternative and promising solution. The other direction can be devoted to the agreements between multiple express companies to use shared lockers to increase the turnover rate. Moreover, we would try and adjust more reinforcement learning algorithms on routing problems to improve the model training stability, achieve better optimal results, and reduce the computational time.

## Figures and Tables

**Algorithm 1:**
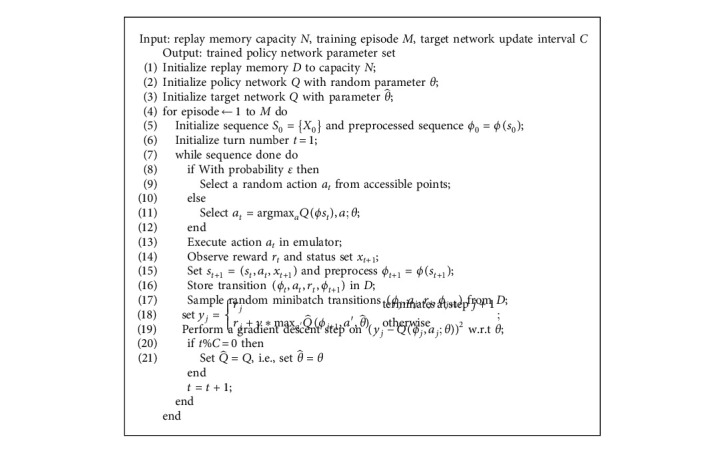
Pseudocode of the routing deep Q-learning algorithm.

**Table 1:** Sets, params, and variables.

*Indices and sets*	
*o* ∈ *O*	Distribution center
*k* ∈ *K*	Trips the courier visits in the planning horizon
*u* ∈ *U*	Express lockers
*s* ∈ *S*	State of parcels
*n* ∈ *N*	Customers request home-delivery
*m* ∈ *M*	Customers using express lockers
*N*	Set of customers request home-delivery
*M*_*u*_^*U*^	Set of customers belonging to locker *u*
*P*	Set of customer nodes
*V*	Set of the distribution center and customers who request home-delivery and locker points

*Parameters*	
*T*_*ij*_	Transport time when a courier visits from point *i* to *j*
*Q*	Capacity of the vehicle
*R*	Capacity of the express locker
FP	Speed of customers who use express lockers to *pickup* parcels from the locker
FD	Speed of customers who use express lockers to *deliver* parcels to the locker
*d*_*i*_^*k*^	Number of parcels the courier *delivers* to point *i* in trip *k*
*p*_*i*_^*k*^	Number of parcels the courier *picks up* at point *i* in trip *k*

*Discrete variables*	
*cd*_*u*,*s*_^*k*^	Number of *s* state parcels customers delivering to locker *u* in trip *k*
*rp*_*i*,*s*_^*k*^	Number of *s* state parcels the courier picking up at point *i* in trip *k*
*rd*_*i*,*s*_^*k*^	Number of *s* state parcels the courier delivering to point *i* in trip *k*
*CU*_*u*_^*k*^	Lost sale when customers want to deliver parcels to locker *u* in trip *k* but fail
*RU*_*u*_^*k*^	Back order when courier wants to deliver parcels to locker *u* in trip *k* but fails
*HU*_*i*_^*k*^	Lost sale when customer *i* requests home-delivery to deliver parcels in trip *k* but fails
*I*_*i*_^*k*^	Number of parcels in the vehicle when the courier arrives at point *i* in trip *k*
*h*_*u*,*s*_^*k*^	Number of *s* state parcels in locker *u* when the courier arrives in trip *k*
*t*_*i*_^*k*^	Arrive time when the courier visits point *i* in trip *k*

*Decision variables*	
*x*_*ij*_^*k*^	Whether trip *k* passes the arc (*i*, *j*)

**Table 2:** Problem parameter values.

	*U*	*N*	*M*	*d*_*i*_^*k*^	*p*_*i*_^*k*^	*Q*	*R*	*E*(FP)^*∗*^	*E*(FD)
Small	3	5	10	[1, 5]	[0, 3]	50	80	0.15	0.05
Medium	5	10	20	[1, 5]	[0, 3]	80	100	0.3	0.1
Large	10	10	20	[1, 5]	[0, 3]	80	100	0.3	0.1

*E*(FP)^*∗*^: expectation of FP.

**Table 3:** Numerical results for small, medium, and large size instances.

Small					Medium				Large			
	Work^*∗*^	Time	Lost sale	Back order	Work^*∗*^	Time	Lost sale	Back order	Work^*∗*^	Time	Lost sale	Back order
1	839786.76	1267.04	114.00	0.00	1654265.36	4594.35	5376.00	0.00	4966772.64	8880.29	31981.00	**762273.01**
2	512958.76	1548.32	281.00	0.00	2032655.12	4918.84	9306.00	0.00	4591076.89	9265.26	27875.00	**74873.33**
3	824524.75	1247.12	197.00	0.00	1819557.32	6034.29	9053.00	0.00	5076658.40	7923.60	25092.00	**52660.04**
4	883975.20	1484.12	224.00	0.00	1841460.66	4586.72	4683.00	0.00	3283198.34	8236.03	26268.00	0.00
5	402729.28	2477.32	239.00	0.00	2034023.22	4609.35	7202.00	0.00	3656831.36	9569.64	27269.00	0.00
6	65456.47	992.66	13.00	0.00	1569743.83	3277.75	704.00	0.00	962931.12	8385.12	11233.00	0.00
7	142611.21	1064.82	99.00	0.00	1844410.22	3528.50	4379.00	0.00	1738881.69	6860.91	2690.00	0.00
8	868373.53	1194.56	335.00	0.00	1376037.66	5733.02	7730.00	0.00	1229802.05	6472.98	1944.00	0.00
9	525973.92	1243.12	252.00	0.00	1378022.45	3536.28	4261.00	**84795.82**	3378352.88	5568.93	12696.00	**77982.19**
10	198619.13	1328.17	67.00	0.00	655985.26	3002.28	643.00	0.00	2295674.02	8173.31	10112.00	**9149.93**
Ave	526500.90	**1384.72**	182.10	0.00	1620616.11	**4382.14**	5333.70	8479.58	3118017.94	**7933.61**	17716	97694
Ave time of each step*∗∗*			**0.001332 s**				**0.001259 s**				**0.001381 s**	
Improvements of time (%)			—				−5.43				9.66	

Work^*∗*^: vehicle inventory × time; Ave time of  each step^*∗∗*^ :  total time/number of step.

**Table 4:** Results comparing for small, medium, and large size instances.

		Ours	State-of-the-art	Traditional RL
Small	Work^*∗*^	450328.51	683152.80	1868115.52
	Time	1442.21	1587.69	4209.10
	Computational time	0.02	14.18	0.02

Medium	Work^*∗*^	3578387.84	2779183.97	10252627.97
	Time	3632.63	3582.39	10951.48
	Computational time	0.08	445.31	0.09

Large	Work^*∗*^	4502386.17	9396333.65	25116312.00
	Time	7256.47	7002.06	21088.15
	Computational time	0.17	5114.21	0.17

Work^*∗*^: vehicle inventory × time.

## Data Availability

The data used in this paper are generated based on our assumptions. The whole project is available at https://github.com/DuYu-BJTU/rl_vrp.git.
